# Proceedings of Societies

**Published:** 1861-04

**Authors:** 


### CHAMPAIGN COUNTY MEDICAL SOCIETY.

Subsequently to preliminary meetings, the following named physicians: John Swain, C. M. Mills, A. J. Crain, C. A. Thompson, J. T. Miller, R. H. Brown, H. C. Howard, S. K. Page, S. W. Kinkaid, W. M. Goodwin, S. L. Bearce and J. F. Isom, met at the office of Dr. Mills, Friday, Jan. 2d, 1858, and organized by adopting a constitution and by-laws, and by electing S. L. Bearce, President; J. T. Miller, R. H. Brown,

Vice Presidents; C. H. Mills, Secretary; S. K. Page, Secretary. The Society now numbers twenty members. Essays have been read as follows: Gun Shot Wounds, by C. H. Mills; Stomatitis Mateina, by A. J. Crain; Pneumonia, by C. A. Thompson; Alcohol, by W. Somers; Cold as a sedatue, by J. T. Miller; Quinine, by R. H. Brown; Remitting Fever, by J. Swain; Rheumatism, by W. M. Goodwin; Tuberculosis, by M. B. Thompson; Croup, by Dr. Mills; Typhoid Fever, by Dr. Goodwin; Hæmaturia, by Dr. Somers; Gelseminam, by Dr. Howard; Cholera Infantum, by Dr. Isom; Diphtheria, by Dr. Mills. At our first annual meeting John Swain was elected President; Vice Presidents, J. F. Isom and D. B. Thompson; Secretary, J. T. Miller; Treasurer, C. H. Mills. At our second annual meeting W. Somers was elected President; Vice Presidents, R. H. Brown and H. C. Howard; J. F. Isom, Secretary.

The Society has undoubtedly been productive of much good. Discussion and interchange of opinion have caused much closer reading than most members have previously been in the habit of.

Urbana, Jan. 4th, 1861.

W. H. GOODWIN, Committee.

### ILLINOIS STATE MEDICAL SOCIETY.

DEAR DOCTOR:

At the last annual meeting of the Illinois State Medical Society, held at Paris, Edgar county, on the 8th and 9th days of June, 1860, the undersigned was appointed Chairman of the Committee on Obstetrics, and Prof. De Laskie Miller of Chicago, Cook county, and J. B. Curtis, M. D., of Decatur, Macon county, Associates.

The Constitution of the Society makes it the duty of this

Committee to prepare an Annual Report on the more important improvements effected in this State in the Obstetric Art, and in the management of diseases peculiar to women and children, and to collect statistics during the year.

To enable the Committee to discharge this important duty, they do earnestly solicit your aid in furnishing the material necessary for such report.

We would direct your attention more particularly, 1st, To the History, Causes and Treatment of Puerperal Inflammations in your locality. 2d. To the Causes and Treatment of Displacements of the Uterus.

Please furnish any information of interest to the profession, relative to the Science or Art of Obstetrics, and Diseases of Women and Children, and forward to the Chairman of the Committee, at latest, by April 20th, 1861. Any matter furnished by you, will be thankfully received and duly credited in our report.

Direct to T. D. FITCH, M. D.,
Kewanee, Henry Co., Ill.,
Chair'n Com. of Obstetrics, Ill. State Med. Society.

---

BOSTON, MASSACHUSETTS, U. S.

The question of the entire immunity from danger which is claimed for Anæsthesia produced by Ether, being still under discussion, the Boston Society of Medical Improvement has appointed the undersigned a Committee "to investigate the alleged deaths from the inhalation of Sulphuric Ether, and to report thereon."

They would therefore request the Medical Profession, or any person into whose hands this may fall, to communicate to either of them such cases, coming within their own observation, as shall serve to this end; giving the place, time and circumstances of their occurrence, with the mode of inhalation adopted, and, especially, information in regard to the following points:

1st—*The kind of Ether used, whether pure Sulphuric Ether, Chloric Ether,* or *Ether Combined with Chloroform.*

2d—*The period after inhalation at which death occurred;* also any other facts which may enable them to form an opinion on the subject of their investigations.

Richard M. Hodges, M. D.,
George Hayward, M. D.,
Solomon D. Townsend, M. D.,
Charles T. Jackson, M. D.

February, 1861.

